# ‘Activating Indigenous ways’ – perceptions of how Australian Indigenous health and wellbeing program evaluations are commissioned and future recommendations

**DOI:** 10.1186/s12939-025-02675-0

**Published:** 2025-11-06

**Authors:** Summer May Finlay, Amohia Boulton, Jenni Judd, Bronwyn Fredericks, Janaya Pender, James A. Smith, Helen Simpson, Yvette Roe, Sophie Kerrigan, Anna Temby, Margaret Cargo

**Affiliations:** 1 University of Wollongong, Wollongong, Australia; 2 University of Newcastle, Newcastle, Australia; 3 Whakauae Research Services Ltd, Whanganui, New Zealand; 4 Central Queensland University, Rockhampton, Australia; 5 The University of Queensland, Brisbane, Australia; 6 Lowitja Institute, Melbourne, Australia; 7 Flinders University, Adelaide, Australia; 8 Charles Darwin University, Darwin, Australia

**Keywords:** Indigenous, Aboriginal and Torres Strait Islander, Evaluation, Commissioning, Policy

## Abstract

**Background:**

In Australia, billions of dollars are spent on Indigenous programs, services, and initiatives annually; however, more evidence is needed regarding which modes of commissioning program evaluations best benefit Indigenous communities. The Australian Productivity Commission called for ‘more and better’ evaluations of Indigenous programs, and commissioning processes that engage Indigenous communities, organisations, and leaders. So, too, have Indigenous representative organisations, Indigenous services, and stakeholders. To date, few studies have sought to characterise the commissioning practices of government and non-government organisations surrounding Indigenous health and wellbeing programs. Fewer still have investigated the role of Indigenous engagement and leadership before, during and after the commissioning process.

**Methods:**

Using Indigenous Standpoint Theory and a qualitative approach, this study illuminates the perceptions of Australian Indigenous and non-Indigenous commissioners, evaluators, and providers on the commissioning of evaluated Indigenous health and wellbeing programs.

**Results:**

Building on a published scoping review by undertaking 23 qualitative interviews with 35 Australian-based participants about commissioning practices, this study verifies and canvases the strengths of individual commissioning models, and the interplay between power, cultural safety, and reciprocity in the commissioning space. The paper also considers the relationship between these three factors along a continuum of practice and posits a sliding scale across the five commissioning models.

**Conclusions:**

To improve the quality of evaluations further attention needs to be paid to the commissioner’s cultural capability. Additionally, Commissioners need to develop their understanding of the relationship between Commissioner and Indigenous people’s power and the utility of evaluations.

**Supplementary Information:**

The online version contains supplementary material available at 10.1186/s12939-025-02675-0.

## Background

In Australia, Aboriginal and Torres Strait Islander people, the Indigenous peoples of the country, continue to experience unacceptable levels of poor health and wellbeing despite successive government policies and programs designed to address this issue [1–3]. While there have been some improvements over time, the current governments’ (state/territory and commonwealth) *Closing the Gap* (CTG) targets reporting demonstrates more needs to be done [1–5]. CTG is a national policy which commenced in 2008 which seeks to achieve health equality between Aboriginal and Torres Strait Islander people and other Australians within one generation (2031) [4]. Given the significant health disparity, evaluations of Indigenous health and wellbeing programs have become increasingly important in assisting governments and Aboriginal and Torres Strait Islander people, communities, and organisations to measure the success of these programs, and better understand what works and why [6–11]. The Australian Government has recognised the critical role evaluation plays in the development of effective policy and programming, as demonstrated by the development of the Indigenous Evaluation Strategy in 2020 (Productivity Commission, 2020). Despite this explicit recognition, there is little understanding of how the actual process behind the commissioning of evaluations impacts upon their utility and use for policy and program development or revision [11, 12].

This research is driven by a call from Indigenous leaders wanting to both influence decision-making processes within the health system and across sectors [13], and to improve the commissioning of health and wellbeing programs to reflect Indigenous needs, priorities, and views on program design, delivery, and evaluation [14]. This push by Indigenous leaders, with the support of non-Indigenous allies, necessitates better designed and evaluated programs that benefit Indigenous populations and achieve the CTG targets [15]. The call for better evaluations is supported by recommendations of the Productivity Commission [16] for government and non-government agencies to work ‘as a partner’ [1] with Indigenous people to ensure that programs, and their evaluations, capture Indigenous interests [17]. Ensuring evaluations are Indigenous-led also aligns with Articles 18–21 and 23 in the United Nations Declaration on the Rights of Indigenous Peoples [18], which outlines the right of Indigenous peoples to act as full and effective participants in the decision-making process that affects their health, economic, and social wellbeing. Indigenous-led commissioning processes also contribute to the aims of Indigenous Data Sovereignty (IDS) movements in Australia, by placing Aboriginal and Torres Strait Islander peoples at the foreground of evaluation data creation, analysis, and dissemination [19]. IDS scholars have repeatedly demonstrated that access to appropriate data is fundamental to First Nations peoples’ abilities to inform better policy and health outcomes [20, 21]. Indigenous-led commissioning of evaluations promotes appropriate custodianship and use of data relating to Aboriginal and Torres Strait Islander people.

Effective Indigenous engagement starts with policy development, the subsequent commissioning of programs, and their evaluation [22]. Commissioning, for the purpose of this research, is defined as *“a dynamic process where decisions are made about allocating funding to address health priority areas and improve health outcomes, which health and wellbeing programs should be delivered to Indigenous populations, and who should deliver and evaluate these programs”* [23]. The commissioning process is guided by supporting frameworks and principles and establishes opportunities for Indigenous engagement in decision-making.

There is an urgent need for research and action at the intersection of the evaluation of Indigenous programs [1, 24] and Indigenous governance [22, 25, 26], particularly in the absence of evidence based on health program effectiveness. Understanding how commissioning practices can enhance Indigenous control and decision-making of Indigenous-specific health and wellbeing programs and their evaluation is necessary, as the gap in knowledge creates impediments to commissioners applying good practices in evaluation tenders of Indigenous health and wellbeing programs. Poor commissioning practices, which lead to evaluations with limited-to-no utility, also impede the development of an evidence base to inform policy decisions on strategic financial investments to improve Indigenous health and wellbeing outcomes. With better-designed programs, evaluations, and improved commissioning practices, there is an increased chance of achieving CTG targets and improving Indigenous health and wellbeing outcomes.

This manuscript aims to strengthen the evidence base on government and non-government commissioning practices of evaluated Indigenous health and wellbeing programs in Australia by building on the findings of a previous scoping review [27]. This manuscript has two specific objectives. First, it explores the Australian alignment with five commissioning models identified (Indigenous-led, delegative, co-design, participatory, top-down) through an internationally focused scoping review (i.e. Australia, New Zealand, Canada, U.S.) examining government and non-government commissioning practices. Second, it identifies the issues, challenges and opportunities related to evaluating Indigenous health and wellbeing programs from the perspectives of Indigenous and non-Indigenous commissioners, service providers and evaluators. This study is the first to focus on stakeholder perceptions of how commissioning models work and the impact the choice of model has on the usability of the findings for service providers and commissioners alike.

## Methods

### Theoretical position

This study was guided by Indigenous Standpoint Theory, which privileges Indigenous voices and subjectivities and recognises that historical knowledge remains prejudiced by Eurocentric or colonial perspectives which restrict Indigenous voices [28–31]. It is research by, with, and for Indigenous peoples, not just ‘about’ [32]. Indigenous Standpoint Theory draws on: Indigenous ontology regarding existence and the nature of reality [32]; an epistemology founded on an Indigenous perspective of knowledge; and axiology grounded in those cultural values; and a methodology influenced by these three factors [31–36]. Throughout this research, Indigenous researchers and the Project Advisory Group (PAG) ensured that reciprocal appreciation for, and understanding of, Indigenous culture and identity was paramount, in both the research questions asked and in interpretations of results [29, 37, 38].

### Methodological approach

This study builds on the findings of a scoping review [27], which identified five commissioning models utilised by government and non-government organisations in contracting Indigenous evaluations for health and wellbeing programs. The commissioning models were characterised as [1] Indigenous-led [2], delegative [3], co-design [4], participatory and [5] top-down (Additional File 1). As outlined in the review, few details of the commissioning practices were reported. The thin description of commissioning practices in the reviewed documents provided a limited understanding of how the models worked in practice and the contextual factors that supported their use. This interview study addressed these gaps to generate a more robust understanding of these commissioning models in an Australian Indigenous context and to identify any models not identified in the scoping review.

### Project governance

As noted above, to privilege Indigenous perspectives, Indigenous Standpoint Theory [39] was used as the theoretical lens in the collection, analysis, and interpretation of the data. The Research Team comprises highly experienced Indigenous (*CI Fredericks, CI Roe, CI Finlay, and AI Boulton*) and non-Indigenous (*CI Cargo, CI Judd, and CI Smith*) investigators. The project staff included non-Indigenous (Simpson, Kerrigan) and Indigenous researchers (Pender). Further Indigenous oversight was provided by the Indigenous members of the PAG which was chaired by Professor Tom Calma.

The PAG supported the research team throughout the entire study, meeting twice a year. This project was largely conducted during the COVID-19 pandemic, therefore, the vast majority of the PAG meetings were held online via video conference. The PAG included: Indigenous and non-Indigenous leaders from senior government and non-government commissioners of evaluation; service providers who receive funding for programs aimed at strengthening Indigenous health and wellbeing; senior advisors with government experience; and professional associations with a mandate to support Indigenous evaluation. Indigenous representation on the PAG privileged Indigenous knowledges and ways of doing in the project. Pre-COVID the PAG, met face-to-face, while during and post COVID the PAG met via video conference.

### Participants

To gain insight into commissioning practices, this study sought the perspectives of three key evaluation stakeholders engaged in the Commissioning of programs by government (federal, state, territory, council) or non-government organisations, (not-for-profit and for-profit). We specifically sought perspectives from: [1] Indigenous and non-Indigenous funders/commissioners [2], Indigenous and non-Indigenous service providers, and [3] Indigenous and non-Indigenous evaluators. Our interest was in the commissioning of health and well-being programs (disease prevention and health promotion programs) that targeted Indigenous people as the primary program participant. We were further interested in stakeholder experiences with the five commissioning models described in the scoping review.

Interview invitations were extended to these three key evaluation stakeholder groups. Participants were purposively sampled based on their known experience with one or more commissioning models. We sought participants who could provide rich information on commissioning practices and contextual factors influencing processes. The research team and PAG initially identified potential interviewees. Consideration was given to experience in Indigenous evaluations, stakeholder spread, geographical representation i.e. national or state/territory and urban, regional and remote locations experiences. Subsequently, additional participants were identified from the interviewees (snowball sampling) and the professional networks of the Research Team and PAG. Contact with participants was made primarily via email. Given the professional connections of the research team, some stakeholders were followed up opportunistically over the phone.

### Data collection

This qualitative descriptive study included in-depth individual, small group (2–4 participants), and focus group interviews (5 or more participants). Interviews were supplemented with a brief demographic survey completed by individual participants. The development of the semi-structured interview guide was led by MC and SMF in collaboration with the team. The interview guide was tailored to the participant’s role: (1) Commissioner (2), Evaluator, and (3) Service Provider. This approach was designed to gain insight into the practical aspects of applying the models, such as timeframes for tender responses, the extent of Indigenous involvement, and the presence of Indigenous policy frameworks or principles. The interview guide was pilot tested on four interviews and revised based on the interviewer experiences.

Commissioners were asked:

- What policy frameworks or principles support the evaluation of Indigenous programs?
- What types of approaches does your organisation use to purchase evaluations of Indigenous health and wellbeing programs?

Evaluators were asked:

We would like you to consider the different commissioning agencies you have been involved with in evaluating Indigenous programs.

- Are the commissioning processes similar?
- If similar, how are they similar?
- If different, how do they differ?

Service providers and evaluators were asked:

- You mentioned [in the question above] the [e.g., co-design] approach. What are its key features?
- Can you describe what the tendering or commissioning process looks like?
- To what extent does this model engage Indigenous leaders, organisations, and communities? At what stage are they engaged? How are they engaged? What are its key features?

Interviewees nominated whether an individual, small group, or focus group interview was the most appropriate in their context. Individual and small group interviews were conducted in person (if feasible for the interviewer and based on COVID restrictions), via video conference (Microsoft Teams, Zoom, or Webex), and over the phone. For face-to-face interviews, interviewees identified a suitable and safe location to share their insights, experiences, and stories on evaluation commissioning. In-person and phone interviews were audio recorded, and video conference interviews were video/audio recorded.

SMF, MC, JJ, and JS conducted the interviews. Following each individual and focus group interview, the researcher completed a contact summary form which noted the overall flow of the interview, duration of the interview, the level of comfort/style and expression of responses provided (e.g. forthcoming with rich responses; tentative in responding), any interruptions or context related issues that may have impacted the interview (e.g. disruptive air conditioner in the room). All interviewees were invited to complete a one-page survey to characterise their experience level, role in supporting evaluation, organisational affiliation, gender, and whether they identified as Aboriginal and/or Torres Strait Islander.

### Data analysis

A professional transcription agency transcribed the interviews after signing a confidentiality agreement. To safeguard interviewees, their organisations, communities, and evaluated programs remained anonymous and confidential, with pseudonyms assigned to interviewees. A set of descriptors replaced organisation names (e.g. Indigenous service provider), communities (e.g. remote community), and programs (e.g. gender-based program) in each of the transcripts. Two researchers (SMF and MC) reviewed the redacted [40] transcripts and returned them to the participants for review and comment. Interviews were analysed using NVivo 20 qualitative software [41].

To address the first objective on the alignment of the five commissioning models within an Australian context, the analysis was guided by a conceptually clustered matrix with the rows reflecting the 13 principles and the columns reflecting the five commissioning models [40]. Each document was coded for the 13 principles as expressed in the models (Additional File 1). A simultaneous coding procedure was utilised such that when a text segment suggested multiple meanings more than one code was applied [42]. For example, sections of text on the principle of Community Context for a particular model co-occurred with codes for ‘time’, relationships’, and ‘trust’. Inductive coding was also used to identify new models or variations to the existing five models and to further identify emergent themes pertaining to the issues, challenges, and opportunities associated with these models.

At the beginning of the coding process, the research team members formed Indigenous/non-Indigenous pairs. Each pair reviewed 2–4 interviews and noted key codes, ideas, and impressions. Each pair discussed their interpretations at the Research Team meetings. Additionally, SMF facilitated three collaborative group-based approaches to data analysis. Codes were further refined by SMF, JJ, HS, and JP. Guided by Indigenous Standpoint Theory, the data was critically examined and discussed more broadly within the group, and the multiplicity of perspectives was accounted for. The credibility of the findings was enhanced through multiple data analyses, peer debriefing with the PAG, progressive subjectivity, and member checking with some participants [37]. This approach also allowed for a detailed understanding of the processes behind each of the models. The results will focus on the processes and highlight which model(s) the processes relate to and the strengths and weaknesses of the processes from an Indigenous perspective.

### Ethical approval and consent to participate

Ethical approval to conduct this section of the research project was provided by the University of Canberra (ethics: 2163), the University of Queensland (2019002659), Central Queensland University (22122), the Aboriginal Health and Medical Research Council (1606/19), Aboriginal Human Research Council of South Australia (04-19-856), and the NT Department of Health and Menzies School of Health Research (2020–3615) Human Research Ethics Committees. Interviewees were provided with the participant information sheet and consent form prior to the interviews. Prior to the interviews being conducted, participants were provided an opportunity to ask any questions before written or verbal consent was obtained. Verbal consent was included in the interview recording.

## Results

We conducted in-depth semi-structured Interviews (*n* = 23) with 35 interviewees as individuals or small groups (*n* = 21) or focus groups (*n* = 2). Most interviewees were non-Indigenous (57%), Commissioners (69%) and from a wide range of Indigenous and non-Indigenous not-for-profit organisations (34% and 31%). However, the role categories (Commissioners, Evaluators, and Service Providers) were not discrete, with many interviewees falling into two categories. 20% [7] of the evaluators are Commissioners, and 2% [6] are service providers. 1% [2] of the service providers are also evaluators (Table 1).

**Table 1:** Role, type of organisation and Indigenous status

Variable	Sub-variable	% (*n*=)
Indigeneity	Indigenous	43% (15)
	Non-Indigenous	57% (20)
Stakeholder type	Commissioner*	69% (24)
	Evaluator*	54% (19)
	Service provider*	29% (10)
Organisation type	Indigenous not for profit	34% (12)
	Indigenous for profit	6% (2)
	Non-Indigenous not for profit	31% (11)
	Non-Indigenous for profit	3% (1)
	State government department	9% (3)
	Commonwealth government department	9% (3)
	University	9% (3)
	Indigenous not for profit	34% (12)
Gender	M	17% (6)
	F	82% (29)
Organisational location	NSW	26% (9)
	QLD	6% (2)
	ACT	0% (0)
	VIC	3% (1)
	TAS	0% (0)
	SA	9% (3)
	WA	0% (0)
	National	54% (19)
Interviewee Remoteness	Urban	63% (22)
	Regional	34% (12)
	Remote	3% (1)
Experience in Indigenous Evaluations (years)	Less than 2	3% (1)
	2–5	23% (8)
	5–10	43% (15)
	10–15	3% (1)
	15+	29% (10)

* Note: The categories are not discrete. Many interviewees fell in two categories. 20% (7) of the evaluators are also Commissioners and 2% (6) are also service providers. 1% (2) of the service providers are also evaluators

This study reinforced the legitimacy of the five commissioning models identified from the scoping review. However, it also identified that these models sit across a continuum with Commissioned evaluations fluctuating between models at different stages of the Commissioning process depending on a range of factors which will be outlined in more detail further in the results. This study further defined how each theme, and 13 good practice practices, are expressed across each of the five models (Additional File 1).

The five models are characterised by three high-order themes: power, cultural capability, and reciprocity. As the scoping review outlines, each higher-order theme is aligned with thirteen sub-themes. This study supports the scoping review findings that the models are not always discrete nor fixed; a single commissioned evaluation can, for different processes, utilise different models. For example, the decision to commission the evaluation could have been made by the Commissioner, with some parameters such as budget and timeframe established which, along with the contracting of the evaluator, would align with the top-down model. However, the Commissioner could require the contracted Evaluator to co-design the evaluation framework with the Aboriginal and Torres Strait Islander service providers, and other relevant organisations such as peak bodies, resulting an evaluation that more approximates a co-design model. The Aboriginal and Torres Strait Islander organisations may be involved in all remaining phases of the commissioned evaluation which could reflect a co-design or participatory model depending on the level of engagement.

### Commissioner, evaluator and service provider perceptions on current commissioning practices

This section of the paper presents Commissioner, Evaluator and Health Service Provider experiences with the commissioning of Indigenous health and wellbeing program evaluations. These experiences and perceptions are encapsulated in six inductive themes (Table 2). The six inductive themes are discussed in relation to the commissioning models to unpack the Commissioning process procedures. The models are bolded and, where appropriate, include the relevant processes in parentheses. There is a strong relationship between the codes and several of the themes. Consequently, where codes are mentioned under an identified theme, they will also be italicised. Quotations are used to support the themes where possible however not every example and every theme will include a quote.

**Table 2:** Inductive codes and definitions

Parent code	Child code	Definition
**Commissioning processes** - The stages of the commissioning of an evaluation from the initial concept to the knowledge translation.	*Conception and/or purpose*	The point of which an evaluation and its purpose is considered.
	*Establishing the evaluation contractual parameters*	Development of a contract for the evaluator that outlines the parameters of the evaluation for example the timeframe, budget and objectives.
	*Designing the evaluation framework*	Includes the process and the final evaluation framework.
	*Contracting the evaluator*	The sourcing, selection, and contracting of an evaluator.
	*Evaluation conduct*	The conduct of the evaluation including the data collection and analysis stages.
	*Evaluation management*	Relates to the Commissioners oversight of the evaluation while it is being conducted.
	*Reporting on evaluation*	The reporting of the evaluation findings.
**Racism**		Prejudice, discrimination, or antagonism by an individual, community, or institution against person, people or organisation because they are Aboriginal and/or Torres Strait Islander
**Time**		The timeframe for identified for part or the entire commissioned evaluation
**Agents of change**		Stakeholders who have the capacity to influence the commission formally or through the evaluation process to better meet the needs of Aboriginal and Torres Strait Islander people.
**Commissioners’ evaluation capability**		The evaluation skills, knowledge and expertise of those who are commissioning evaluations.
**Funding**		Relate to the funding committed to the evaluation

### Commissioning processes

Evaluators and Services Providers largely discussed the commissioning practices of Government Commissioners. The processes that were identified as having the most influence on the conduct of the evaluation and, therefore, the utility of the findings by and for Aboriginal and Torres Strait Islander people were the initial phases, which include the:

- Conception of the evaluation and outline of the purpose.
- Establishment of the evaluation parameters.
- Design of the evaluation framework.
- Contracting of the Evaluator.

The other processes were identified as:

- Conduct of the evaluation.
- Evaluation management.
- Evaluation reporting.

The results will focus on the initial four phases of the commissioning process given the significant impact they have on the evaluation itself, with the final three phases described only. Interviewees discussed **top-down** and **participatory** commissioning most often and to a lesser extent delegative and Indigenous-led models however they were described by participants as the least preferred by most Commissioners, Evaluators, and Service Providers.

**Fig. 1:**
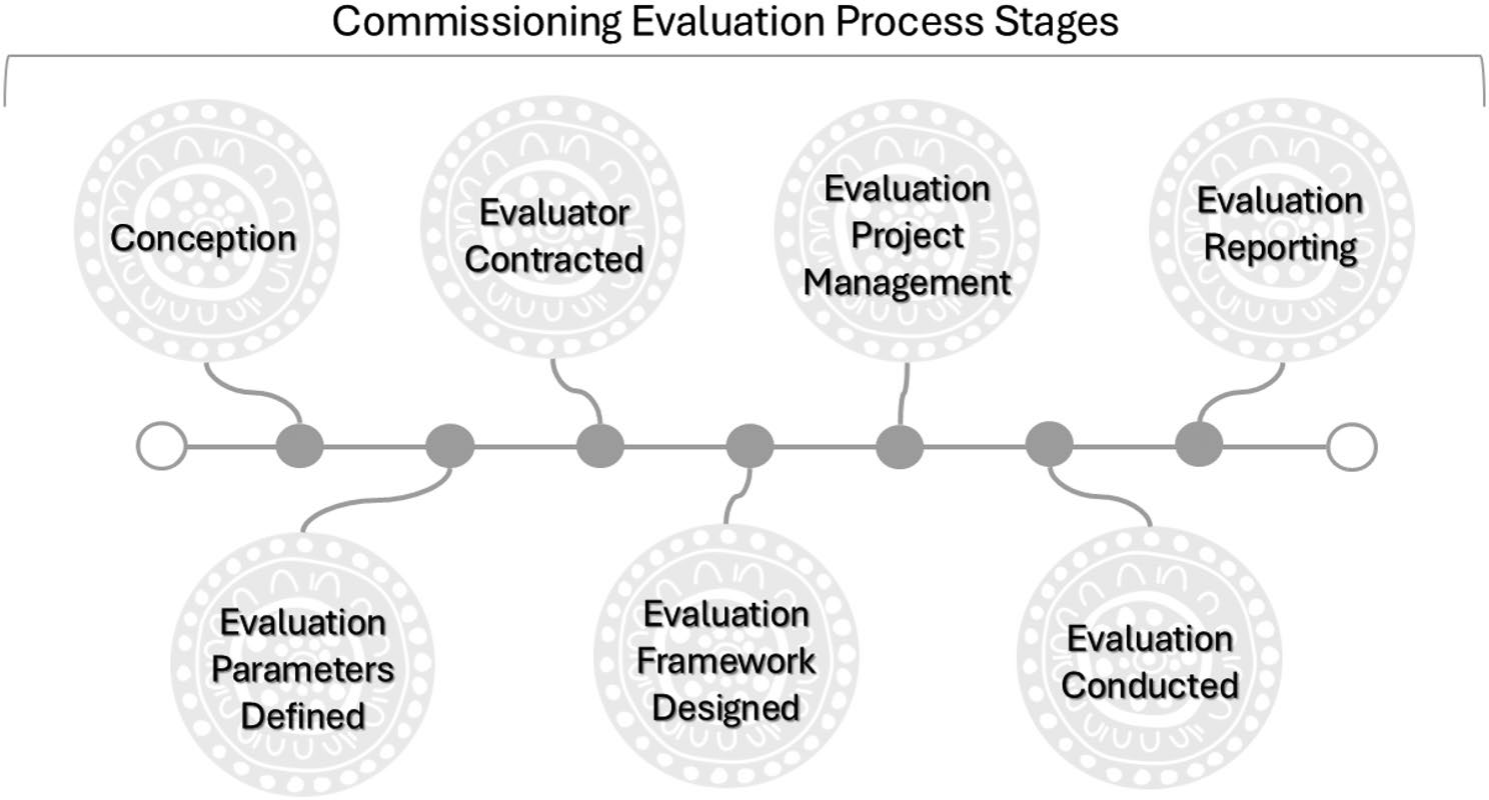
Stages of commissioning Indigenous health and wellbeing evaluations

#### Conception and/or purpose

Commissioners, Evaluators, and Service Providers pointed out that the Commissioner typically identified programs to be evaluated and often conceived an evaluation for their own purposes (**top-down**):

Sometimes if a program is ending, it is good to have an evaluation completed by October/November because if you do need to do a bid for government for new or continued funding. (Ginny, Indigenous Commissioner, government department)

All three stakeholder groups, except for one not-for-profit Commissioner, described little input from Indigenous people at this stage and rarely, if ever, by the service providers (**top-down and participatory**). Where there is Indigenous input into the commissioning process, it is often a Commissioner employee, whose views may be impacted by employer obligations. Therefore, the power is still maintained by the Commissioner (**participatory)** serving the Commissioner’s interest with little to no benefit for the service providers.

#### Establishing the evaluation contractual parameters

All stakeholders discussed how the Commissioner, particularly government Commissioners, often sets the parameters such as the timeframe, budget, aims, and objectives (**top-down**). Evaluators and service providers noted that little consideration was given to how these parameters would impact the service providers engaged in the evaluation (**top-down**). *Time* and *budget* parameters, usually set at this stage, were identified as barriers or enablers to a project benefiting Aboriginal and Torres Strait Islander people. Both themes are discussed later in the results.

One participant noted that engaging the service providers in the development of an evaluation framework – the document that guides the evaluation process including data collection and analysis – is vital to “really achieve some insightful long-term solutions” (Wallander, non-Indigenous, Indigenous not-for-profit). They also demonstrated that Service Provider input ensured an evaluation would be fit for purpose for both the Service Providers and Commissioners.

What looks picture perfect on paper may cause so much delays and so much heartache and headache for the departments and Commonwealth themselves

Some non-government Commissioners worked closely with service providers to co-design the evaluation, ensuring that the Commissioner and Service Providers’ needs were met throughout the process. This provides the additional benefit of *two-way learning*, which increased the knowledge and skills of both stakeholders.

#### Designing the evaluation framework

Commissioners and Evaluators noted that the design stage was more variable; it may be a part of the evaluator contract, can be a separate process the Commissioner outsources, or the Commissioner can develop the framework internally. Regardless of the approach, evaluators noted an *evaluation capability* deficit in some, particularly government, Commissioners. This lack of *evaluation capability* leads the Evaluator to act as a *change agent* by upskilling the Commissioner on good practice evaluations. For example, a Request for Quote (RFQ), the document Commissioners provide to potential suppliers seeking a quote for the discrete piece of work, might call for a **co-design** approach. The project budget, *timelines*, or expected *engagement* with Aboriginal and Torres Strait Islander people, however, may not allow for genuine **co-design**, resulting in a **participatory** model.

If the evaluator develops the evaluation framework, Indigenous engagement in the framework can either be required by the Commissioner or dependent on the Evaluator. Participants noted three differences in the type of Indigenous engagement;

1. The Evaluators are Aboriginal and Torres Strait Islander people (**participatory**).
2. The Evaluators (Indigenous or non-Indigenous) work with relevant Aboriginal and Torres Strait Islander people or organisations (**participatory or co-design**).
3. The non-Indigenous Evaluator develops the framework independently of Aboriginal and Torres Strait Islander people (**top-down**).

When a non-Indigenous commissioning organisation develops the framework, there can be a lack of appropriate Aboriginal and Torres Strait Islander engagement (**top-down and participatory)**. One government commissioner described a cultural panel established to provide guidance to Commissioners, which included Aboriginal staff who had a “particular interest and, to some extent, a stake in that particular program” (Michael, non-Indigenous government Commissioner). Another government Commissioner stated that the “standard process would be for us to design and sign off on five or 10 questions and then fund a consultant” (Ginny, Indigenous government Commissioner). Several Evaluators stated that where a government Commissioner required Aboriginal and Torres Strait Islander input into the design of the evaluation framework, it was usually done through an Aboriginal and Torres Strait Islander Reference Group. Other Commissioners and Evaluators discussed a **co-design** approach, however, which of these parties initiated the **co-design** approach varied between the Commissioner and the Evaluator. Evaluators and Service Providers also described that often the term **co-design** was used by Commissioners, when the approach aligned with a **participatory** model due to the power being maintained by the Commissioner. One not-for-profit, non-Indigenous organisation allowed the funded service provider to determine which approach they would prefer (**co-design or delegative**). Another, a government Commissioner, determined that the Evaluator would be employed (**top-down**) to **co-design** the evaluation framework with relevant Aboriginal and Torres Strait Islander stakeholders.

#### Contracting the evaluator

The selection of the Evaluator is an administrative step, in essence, that has to be done through a process that is correct within the bureaucratic and regulatory system. (Sandy, non-Indigenous Government Commissioner)

Commissioners and Evaluators detailed how the Commissioner usually contracts an Evaluator “in-house” or, at times, involves relevant stakeholders as determined by the Commissioner (**top-down**,** participatory**, **or co-design**). They also described the selection of an Evaluator, starting with an RFQ, which included questions relating to the **cultural capability** of the evaluating team. One Evaluator reported that the **cultural capability** of Evaluators had recently increased in importance in responding to RFQ criteria:

(**cultural capability** of the evaluator is) very important, so when it comes to responding to criteria, there’s often sections that talk about how is what we do culturally safe, how do we engage in a culturally safe way and recently we’ve even had commissioners asking for roles percentages of Aboriginal staff to make sure that it’s not tokenistic. (Sarah, For Profit non-Indigenous Evaluator)

While questions relating to cultural safety are asked in RFQs, Evaluators and Commissioners noted that there is no consistent approach to assessing the criteria and no requirement for an Aboriginal and Torres Strait Islander person to be involved in the selection process. A participant described a **top-down** approach and noted that they “may or may not get an Aboriginal bureaucrat on that assessment panel” to provide an Indigenous perspective on the suitability of a potential contractor. The weighting given to cultural safety “really depends on the person’s judgment” (Ginny, Indigenous government Commissioner) and is a “trade-off between capability, availability, and all kinds of capability, technical as well as cultural capability” (Sandy, government non-Indigenous Commissioner). Commissioners stated that the Service Providers of evaluated programs are not usually directly engaged in the selection process (**top-down and participatory**), however, they may be consulted (**participatory**) on potential Evaluators.

#### Evaluation conduct

The conduct of the evaluation relates to the data collection and interpretation. Indigenous engagement in this phase is usually determined by the contract between the Commissioner and the Evaluator, and by the Evaluator themselves. For example, the evaluation can be overseen by an Aboriginal and Torres Strait Islander reference group, who may be involved in the decision making (**co-design)** or merely informed of project updates (**participatory**). Aboriginal and Torres Strait Islander people can be, though not always, involved in the data collection and interpretation, and depending on their ability to influence these processes, their engagement could align with the **co-design**, **participatory** or **top-down** models. For example, if they are involved in how the data will be collected, solutions to emerging issues and the interpretation of the data that aligns with the **co-design** model. Whereas if they are tokenistically engaged as mere interviewers in a qualitative study, without any influence of the data collection design or interpretation of findings, this approach aligns with the **top-down or participatory models**.

#### Evaluation management

The evaluation management process relates to the working relationship between the Commissioner and Evaluators, including contracted meetings. The aim of these meetings is to allow the commissioner to stay informed of the evaluation progress and to collaboratively problem-solve emerging issues or variations of the evaluation framework. Where a Commissioner was dominant and directive, and lacked *flexibility*, this reflected **top-down** model, however, if they engage with the Evaluator in a way that demonstrated respect and space to advocate for the Aboriginal and Torres Strait Islander people and communities (*agents of change*) this aligns with **participatory** or **co-design models**, dependent on where final decision-making rests.

#### Reporting on evaluation

The reporting of evaluation findings can take many forms and is usually determined by the Commissioner. This could include who will receive the findings beyond the Commission (participants, relevant Communities or other stakeholders) and the extent and timing of publications. A Commissioner’s refusal to release the report, a selected release, or the release of a summarised version would suggest a **top-down** approach. If the Commissioner engages with the Aboriginal and Torres Strait Islander people and organisations involved in the evaluation on what to release, this would be considered either **participatory** or **co-design** depending on who makes the final decision.

### Racism

Racism was both explicitly identified by stakeholders as an issue in the commissioning process, as well as being implicit in participant’s responses. Racism was often described as a lack of **trust** in Aboriginal and Torres Strait Islander people and organisations to engage in an evaluation in an unbiased way, contrasted with the presumption that non-Indigenous people could be impartial. For this reason, several of the Commissioners indicated that Aboriginal and Torres Strait Islander Service Providers were not included in the development of the RFQ, design of evaluation parameters, or the selection of an evaluator. Some Commissioners did indicate a desire to engage Aboriginal and Torres Strait Islander people in a** co-design** approach in the initial commissioning stages, or even **delegate** the evaluation, but encountered resistance at more senior levels, resulting in a **top-down** or **participatory** approach. There was also a perception from Evaluators and Service Providers that some Commissioners lacked *cultural capability* and understanding of the heterogeneity of Aboriginal and Torres Strait Islander people. Some Evaluators reported that Commissioners questioned the necessity of travel to remote communities during data collection, believing data from less remote regions could be representative of all Aboriginal and Torres Strait Islander people.

### Commissioners evaluation capability

Several participants discussed the need for Commissioners to demonstrate better understanding of good practice for evaluations. Commissioners make decisions across the entire evaluation process, from conception, through to the development of a RFQ, the selection of an Evaluator, and engagement with the Evaluator during data collection. All stages of this process have the potential to impact the utility of an evaluation. Therefore, it is vital that Commissioners have a good understanding of evaluation to make informed decisions. Improved Commissioner evaluation capability would ensure that the parameters they set for an evaluation are fit for purpose, and that they engage the right Evaluator. When interviewed, some Evaluators and Service Providers detailed how a lack of in-depth understanding of evaluations led Commissioners to make decisions that are not aligned with good practice, or of benefit for Aboriginal and Torres Strait Islander people. Once participant stated that Commissioners *“lack of knowledge experience and grounding that will be able to make that evaluation accurate and useful and effective for us”* (Ralph, Indigenous Service Provider).

One Evaluator interviewed described instances of evaluations being undertaken by someone who “*doesn’t even have proper expertise in evaluation*” (Sarah, Indigenous Evaluator) because the Commissioner themselves lacked the *evaluation capability* to understand what was required. The lack of *evaluation capability* means that Aboriginal and Torres Strait Islander people and Service Providers have a problem getting:

Commissioning entities to understand what we really want, clarity around what the scope is, clarity around even the process that they should be going through to get information to do an evaluation and ensuring that they get the right data. (Ralph, Indigenous Service Provider)

There are demonstrable impacts of this lack of* evaluation capability* on the utility and usefulness of evaluations undertaken, resulting in evaluations, and Commissioners, that aren’t able “to, you know, come up with some good solid stuff that can inform decision making moving forward”. (Cara, Indigenous Evaluator). The influence of a Commissioner’s *evaluation capability*, and their ability to either enhance or limit an evaluation, is further demonstrated and expanded on in the following themes; time, agents of change, and funding.

### Time

In most of the evaluations discussed by participants, *time* was seen as a barrier to conducting quality evaluations that *benefit* Aboriginal and Torres Strait Islander people. Participants described narrow response times to an RFQ or to conduct the evaluation, due to the lack of a Commissioner’s *evaluation capability*, or Commissioner-driven priorities; either the Commissioner lacked the understanding of how a limited timeframe could impact an evaluation, or internal processes and directives – often by more senior people, including the Minister – were prioritised. The small amount of *time* evaluators had to submit a response to RFQs meant that they were “rarely” able to *engage* with Aboriginal and Torres Strait Islander people and organisations prior to submitting. Evaluators described their inability to engage with the service providers as limiting their capacity to design an evaluation framework that would be tailored to the *context* of the individual service providers. They were also unable to *engage* other Aboriginal and Torres Strait Islander people or organisations with relevant knowledge and expertise with whom they did not already have a *relationship* or could only manage superficial engagement.

The limited *time* Commissioners gave Evaluators to conduct an evaluation also limited the evaluator’s capacity to undertake a rigorous evaluation, reducing the utility of the findings because “you can’t do a decent job when you’ve got 2 minutes to do it” (Harry, Indigenous Service Provider). Commissioners were often identified as being unaware of the limitations of their *time* requirements due to a lack of *evaluation capability*. For multi-jurisdictional or jurisdictional-based evaluations, given the heterogeneity among Aboriginal and Torres Strait Islander people, communities, and organisations, several Evaluators and Service Providers interviewees stated that it was essential to *engage* widely. Service providers and evaluators noted that the unique needs of Aboriginal and Torres Strait Islander communities and organisations often cannot be factored into the evaluation due to limited *time*, reducing the *cultural safety* of the evaluation and the utility for policymakers and Aboriginal and Torres Strait Islander people alike.

Commissioners setting limited timeframes meant the required *relationships* and *trust* could not be developed because “there’s no time for engagement” (Asil, Indigenous, Indigenous Service Provider). The impact of this lack of *relationship* or *trust* between Evaluators and Service Providers was that they were disinclined to engage in the evaluation because “unless trust is there, I don’t think they (Service Providers) … will really open to anyone external” (Wallander, non-Indigenous, Indigenous not-for-profit). At times service providers were contractually required, or felt obliged, to maintain a *relationship* with the Commissioner due to the *power* imbalance, regardless of whether the evaluation was *culturally safe*, of *benefit*, or adequately *engaged* them in the design, further eroding the *trust* Service Providers had in the Commissioners. *Time* also limited the evaluators’ capacity to travel to remote locations which meant the findings could not be generalisable to these communities. One Indigenous Service Provider and Evaluator said it takes…

a lot longer to survey remote communities…and that brings with a whole lot of time considerations that may not be apparent to the funding agent

Unfortunately, the lack of time meant that evaluations sat at the **top-down** or **participatory** end of the spectrum; conversely, “making time” meant that evaluations were more likely to include elements of **co-design**.

Across the board, time is a key factor to the success of Commissioned evaluations. It has an impact on a range processes across the Commissioning spectrum. It also as a significant impact on how good practice evaluation principles are undertaken.

### Agents of change

For **top-down** and **participatory models**, Evaluators and Service Providers reported that evaluators acted as potential *agents of change* in commissioning. Evaluators, due to being contracted by Commissioners, described having less *power* than the Commissioner, however, due to their skills and expertise, they felt able to influence the commissioning process, and compensate for a Commissioner’s lack of *evaluation capability*. For example, in some instances, Evaluators argued that more *time* or *budget* was required to meet the evaluation aim and objectives:

I was able to then negotiate with the funders and the contract managers at the time, to gift me more time to actually do a community consultation. (Asil, Service Provider and Evaluator)

Similarly, if the Evaluator believed that the evaluation aims and objectives did not align with what they perceived to *benefit* for Aboriginal and Torres Strait Islander people or did not suit with the *community context*, they suggested changes to the Evaluation Framework:

I would say most of the time if we put a strong case forward that things need to be done differently or there needs to be some flexibility, most Commissioners are open to taking some of that on board. It’s not always an easy process. Sometimes, if they’ve got preconceived ideas or timelines, are often a really big issue for us that we want to engage Community and people and organisations properly, and that takes time. (Sarah, non-Indigenous Evaluator, Indigenous for-profit organisation)

In these situations, evaluators shifted the evaluation from **top-down** or **participatory** towards **co-design**.

### Funding

Budgets were a theme discussed by all stakeholder groups as having either a positive or negative impact on the evaluation. Participants described evaluation budgets as largely determined by the Commissioner. Some noted that Commissioners with limited *evaluation capacity*, little understanding of the program being evaluated, and the *community context* in which the program is being evaluated often do not allocate enough *time* or *funding* for the evaluation. One evaluator described funding as an issue because

there’s no opportunity really to upskill the broader community around evaluation or the purposes of evaluations and how the, how their say in that evaluation actually impacts on, you know, on the process. (Hermione, Indigenous Evaluator)

Several evaluators described difficulties in interpreting the depth and rigour expected by a Commissioner when the RFQ did not indicate a budget range:

I guess a big challenge we have often is that we don’t know what the budget is, it’s rare to know what a cap is, and so we’re sort of … we always go with what we think is best practice. (Sarah, non-Indigenous, Indigenous not-for-profit)

Evaluators detailed how the size of the budget dictates how many communities can be *engaged*, how many visits to each community could be made, and the ability to travel to remote communities. Evaluators and service providers noted that a *budget* that limits the number of communities or service providers in the evaluation reduces the generalisability of the findings. Similarly, a small budget limits evaluators’ capacity to develop *relationships* and *trust* with communities and Service Providers; therefore, it cannot be conducted robustly and *culturally safe*. Some Evaluators believed that Commissioners were only sometimes aware that limiting the project budget could limit the usability of the findings.

## Discussion

This study has detailed the myriad and complex processes that are often used without consideration of the impact they have on the utility of the evaluation findings. In identifying and codifying the commissioning practices into models, Commissioners will be able to consciously make decisions on how they commission Aboriginal and Torres Strait Islander health and wellbeing evaluations. Furthermore, evaluators and Aboriginal and Torres Strait Islander people will be able to use the models and their definitions to critique current commissioning practices to hold Commissioners to account.

Interviews reflect the overwhelming experience of **top-down** and **participatory** commissioning of Indigenous health and wellbeing evaluations, despite these practices being the least preferred by most stakeholders. Evaluators and Service Providers demonstrated that these practices limited the useability of the evaluations for program and policy development and tended to be culturally unsafe for Aboriginal and Torres Strait Islander people. The commissioning practices most widely discussed were that of governments (federal, state/territory and local), due to the dominance of government funding in Aboriginal and Torres Strait Islander health and wellbeing.

### Evaluation capability

Evidence-based policy is a fundamental global driver of the policy cycle [43–50], with evaluation a key aspect of developing the required evidence [8–10, 44, 51–54]. Despite the utility of program and policy evaluation, Commissioners often need a better understanding of evaluation and the impact their decisions (or lack thereof) have on the utility of the evaluation findings for program and policy development. This is largely because the drivers in the early stages of the commissioning process are not related to the intended outcomes, outputs, or cultural safety but are based on the Commissioners’ needs or priorities.

Globally, good practice Indigenous evaluations [14, 52, 55–65] including those relating to Aboriginal and Torres Strait Islander people [16, 61, 65–67], demonstrate principles associated with stakeholder engagement, relationships, understanding community context, cultural safety, and benefit to all stakeholders. However, the Commissioners often do not take into consideration some or all the Indigenous best practice evaluation principles. Alarmingly, Commissioners were often not aware of the significant limitations they place on an evaluation, such timeframes and budgets that restrict evaluators and service providers ability to design a culturally safe evaluation that reflects the purpose of the program being evaluated. Therefore, reducing the usability and generalisability of the findings for Aboriginal and Torres Strait Islander people and organisations.

At a time when billions of dollars are being spent in Australia on improving the significant health disparities Aboriginal and Torres Strait Islander people experience [68–71], more rigorous evidence is required to inform policy and practice [6, 49, 72]. To achieve the goal of evaluations that can truly be used to inform policy and practice, Commissioners, in particular Australian state and territory governments, need to have greater evaluation capability to understand what good practice commissioning entails and how to undertake those practices, such as ensuring adequate time and budget through all stages of the commissioning process. Additionally, Commissioners need to label evaluation practices with the correct methodological terminology, and avoid the misuse of terms, such as ‘co-design’, that may contribute to these terms becoming buzz words [73]. Many Evaluators and Service Providers raised concerns that participatory practices are being labelled as co-design without truly understanding their differences. Mislabelling a methodology breeds distrust in future projects using this methodology, even if their process aligns with the true intent of co-design.

### Time and budget

The timeframes for responses to an RFQ or the conduct of an evaluation need to be sufficient to ensure that the evaluation framework and the evaluation sufficiently engage with Aboriginal and Torres Strait Islander people. Regardless of the size of the project, developing and maintaining relations with Aboriginal and Torres Strait Islander people to ensure that a research project is conducted in a culturally safe way and is of benefit to Aboriginal and Torres Strait Islander people is vital. Evaluation guidelines and frameworks across the country clearly articulate this need [10, 61, 65–67]. A budget must enable the relationships with and input by the appropriate Aboriginal and Torres Strait Islander people in a way that is deemed sufficient by Aboriginal and Torres Strait Islander people. It is only through the adequate provision of a timeframe and budget that enables Aboriginal and Torres Strait Islander engagement that culturally safe and community benefit can be achieved.

### Indigenous Data Sovereignty

Good engagement with Aboriginal and Torres Strait Islander people should align with Indigenous Data Sovereignty principles [74–77]. The key aspects of Indigenous Data Sovereignty (IDS) being that Indigenous communities have the right, skills, and understanding of their communities to determine why, how, and for what benefit data should be collected [74–77]. The best available evidence demonstrates that Aboriginal and Torres Strait Islander people, communities and organisations need to be engaged in the development of policies that impact them to see outcomes for the Community [6, 67]. Australia, as a colonial country, has a history of paternalism and marginalisation of Aboriginal and Torres Strait Islander people, much of which has been driven by racism [78]. This in part has driven the IDS movement globally that has led to the development of IDS principles. Indigenous people globally have been advocating for their knowledge systems to be valued as equal to Western knowledge systems [79–81]. The advocacy has also centred around their self-determination, a core principle underpinned in the United Nations Declaration on the Rights of Indigenous People [82], which Australia signed. This advocacy promoting Indigenous knowledges and self-determination has taken many forms. In relation to data, it has manifested in the IDS movement. IDS is:

the right of Indigenous peoples to determine the means of collection, access, analysis, interpretation, management, dissemination and reuse of data about the Indigenous peoples from whom it has been derived or to whom it relates. Indigenous data sovereignty centres on Indigenous collective rights to data about our peoples, territories, lifeways and natural resources(77 pg. 1)

IDS has not been at the core of commissioning practices by non-Indigenous Commissioners though there has been a recognition of the movement more recently with the publication of the Commonwealths Framework for Governance of Indigenous Data Practical guidance for the Australian Public Service [83]. There is much data collected on Aboriginal and Torres Strait Islander people, however, there is little that is collected explicitly for Aboriginal and Torres Strait Islander people [79–81]. This includes data collected as part of an evaluation, although the programs being evaluated are funded and designed to improve the health and wellbeing of Aboriginal and Torres Strait Islander people. As outlined above, what is required is for Commissioners to have an understanding and respect for IDS principles to ensure that IDS principles are embedded into each commissioned evaluation. This requires a fundamental shift in how many Commissioners operate, putting Aboriginal and Torres Strait Islander people’s needs above their own.

To improve the quality, rigour and benefit of evaluations, Commissioners need to have greater evaluation expertise and understanding of Aboriginal and Torres Strait Islander people and communities to ensure that evaluations are fit for purpose for their organisation and Aboriginal and Torres Strait Islander people. Fundamentally, what’s required is a shift in whose needs are prioritised and a recognition that the Commissioner, while holding power, should be deferring to Aboriginal and Torres Strait Islander people.

### Limitations and strengths

#### Limitations

Most interviewees were Commissioners, with only a few Aboriginal and Torres Strait Islander service providers. While all stakeholder types were represented (Commissioners, Evaluators and Service Providers) it was not possible to ensure that there was a comprehensive geographical spread i.e. state/territory/national and urban regional and remote. For many participants, however, their geographical location does not reflect their geographical experience. A number of participants operate nationally, and therefore their experience covers multiple states and territories, as well as areas of varying population density. For Aboriginal and Torres Strait Islander participants, their physical location also does not limit the extent of their connection to other regions, or their knowledge and experience. Family, community, and cultural connections extend beyond areas currently delineated by state or territory borders. These factors ensure a broad experience is represented amongst participants, despite a perceived lack of geographical spread. The study was conducted during the COVID-19 pandemic, therefore, while face-to-face was the preferred method, its use was limited.

#### Strengths

There were numerous study strengths, such as openness and honesty expressed in the interviews, allowing participants to identify the type of interview, multiple coders and group coping process, and Indigenous leadership. The experience of the interviewers both as researchers and evaluators in the Aboriginal and Torres Strait Islander space increased their credibility therefore interviewees appear to respond with openness and honesty which increased the utility of the findings. Allowing interviewees to identify how they would like to be interviewed also aided in providing a level of comfort that increased open and honest responses. It is also noted that there were few descriptions on the Indigenous-led and delegative models; this could be because there is often little opportunity for Aboriginal and Torres Strait Islander organisations to commission evaluations independently of their funder due to limited finances, Moreover, few Commissioners use a delegative model. There was, however, widespread support among Service Providers and Evaluators for the Indigenous-led model. Finally, the Indigenous leadership on the project ensured that the findings broadly reflected the needs, perceptions and values of Indigenous people.

## Conclusion

Evaluations can improve policy and programs for Aboriginal and Torres Strait Islander people if designed and conducted well. Unfortunately, many evaluations fall short of good practice because Commissioners lack an understanding of how their decisions impact an evaluation’s utility. To improve the quality, rigour and benefit of evaluations, Commissioners need to have greater evaluation expertise and understanding of Aboriginal and Torres Strait Islander people and communities to be able to ensure that evaluations are fit for purpose, not just for their organisation but also for Aboriginal and Torres Strait Islander people. A key aspect of achieving this is adherence to IDS Principles. While Commissioners make attempts to ensure that Cultural Safety is considered in the Commissioning processes, often their own needs are placed above that of Aboriginal and Torres Strait Islander people. More needs to be done to ensure that Commissioners, particularly non-Indigenous Commissioners, have the evaluation and cultural capability to assist in closing the health inequality gap.

## Supplementary Information

Below is the link to the electronic supplementary material.

Supplementary Material 1

## Data Availability

No datasets were generated or analysed during the current study.

## Abbreviations

CTG: Closing the Gap
IDS: Indigenous Data Sovereignty
PAG: Project Advisory Group
RFQ: Request for quote
