# Supplementary material for: ‘Activating Indigenous ways’ – perceptions of how Australian Indigenous health and wellbeing program evaluations are commissioned and future recommendations
Source: Int J Equity Health. 2025 Nov 6;24:303. doi: 10.1186/s12939-025-02675-0 (PMC12593848; doi:10.1186/s12939-025-02675-0)
Supplement: Supplementary file 1 — Supplementary Material 1 [file 12939_2025_2675_MOESM1_ESM.docx]

Additional File 1

|  | **Top-Down** | **Participatory** | **Co-design** | **Delegative** | **Indigenous-led** |
| --- | --- | --- | --- | --- | --- |
| **Power** |  |  |  |  |  |
| Indigenous engagement across all stages of the commissioning (conception, contracting and management) | Limited engagement by Commissioner, if present, often tokenistic. Commissioner does not ensure the evaluator engages with Indigenous communities. | Varied participation initiated by Commissioner from tokenism consultation to co-design | In principle high level of engagement | High Indigenous engagement in the commissioning process –Indigenous organisation delegated authority to administer funding and to identify external evaluator by govt agency or to support internal evaluation | Indigenous leadership in the commissioning process – impetus for commissioning, funding source and management/ administration of commissioning completely by Indigenous organisation |
| Partnership between Indigenous and non-Indigenous stakeholders across all stages of the commissioning (conception, contracting and management) | Not referred to | Commissioners can initiate partnership but often does not. Where it is present there can still be one-way accountability to funder | Presence of a partnership with shared decision making between Commissioner and Indigenous people. The Commissioner expects co-design to occur between evaluators and the Indigenous community | Partnership, if present, are initiated by Indigenous commissioners who also expects it from the evaluators | Not applicable |
| Self-determination | No opportunities for self-determination present in the commissioning of the evaluation | No opportunities for self-determination present in the commissioning of the evaluation | No opportunities for self-determination present in the commissioning of the evaluation | Self-determination is a key aspect of a delegative model in the commissioning of the evaluation; responsibility delegated to Indigenous people to make decisions | The commissioning process is by Indigenous people for Indigenous people |
| Accountability | One-way accountability to funder | In principle there is mutual accountability between the Commissioner and Indigenous people however often is one-way accountability to funder | In principle there is mutual accountability between the Commissioner and Indigenous people however there can still be one-way accountability to funder. In principle the Commissioner expects mutual accountability between the evaluator and Indigenous community | High level of accountability between the Commissioner and the Indigenous community. The Commissioner also expects mutual accountability between the evaluator and Indigenous community | Tangible and intangible accountability to the Indigenous community is prioritised over accountability to the funder |
| **Cultural safety** |  |  |  |  |  |
| Cultural capability | Limited cultural capability in commissioner; evaluator may or may not be culturally capable | Limited cultural capability in commissioner; evaluator may or may not be culturally capable | Commissioner and evaluators tend to have more cultural capability | High cultural capability of commissioner. Evaluator culturally capable due to identification by Indigenous community | High cultural capability of commissioner. Evaluators are culturally capable. Supports internal evaluation of funded Indigenous organisations |
| Culturally responsive | Commissioner generally non-responsive to cultural protocols and does not ensure the evaluator is culturally responsive | Commissioner often non-responsive to cultural protocols | Commissioner often responsive to cultural protocols under the guidance of Indigenous partners and expects evaluators to do the same | Commissioning process often guided by Indigenous strategic framework, underpinned by Indigenous values and cultural protocols. Evaluators are expected by Commissioner to also adhere to Indigenous values and cultural protocols | Commissioning process guided by local Indigenous strategic framework, underpinned by Indigenous principles and cultural protocols |
| Respect | Commissioners do not privilege processes that make the Indigenous community feel valued nor do they ensure that the evaluators make the Indigenous community feel valued | Commissioners may privilege processes that make the Indigenous community feel valued depending on level of Indigenous engagement | Commissioner often responsive to cultural protocols that make the Indigenous community feel valued under the guidance of Indigenous partners and expects evaluators to do the same | Commissioner responsive to cultural protocol and privileges processes that make the Indigenous community feel valued under the guidance of Indigenous [partners]. Evaluators are expected by Commissioner to also make the Indigenous community feel valued under the guidance of Indigenous [partners]. | The Commissioning is done from an Indigenous standpoint which prioritises local aspiration, views and cultural protocols that demonstrate genuine value for Indigenous communities |
| Community Context | Commissioners do not understand the community and its needs and does not foster the expectation that community context be built into the evaluation | Commissioners often do not understand the community and its needs and does not foster the expectation that community context be built into the evaluation | Through the partnership Commissioners often understand the community and its needs and expects the evaluators to do the same | Commissioning is driven by Indigenous identified priorities, needs or aspirations and expects the same from identified evaluators | Commissioning is driven by Indigenous identified priorities, needs or aspirations of the individual community |
| Trust/ relationship building | Absent | In principle there is trust/relationship building however often is limited or absent | Trust/relationship building is key to co-design | Commissioner recognises the importance of trust in the evaluation and invites the Indigenous community to either conduct the evaluation themselves or identify an evaluator | Commissioning process builds and maintains trust with Indigenous community from the conception of the program its evaluation and beyond |
| **Reciprocity** |  |  |  |  |  |
| Community benefit | Not a priority of the Commissioner to return results to benefit community | Community benefit varies depending on level of participation of Indigenous people | Community benefit is key element of co-design which is honoured by the Commissioner which also expects the evaluator to honour. | Commissioning of the evaluation prioritises community benefits | Commissioning of the evaluation prioritises community benefits |
| Hospitality/ generosity | Commissioner has a tokenistic understanding of hospitality/generosity and may something transactional such as a voucher. Commissioner does not ensure the evaluators offer hospitality/generosity though the evaluators may do this of their own accord | Varies from tokenistic to genuine hospitality depending on level of participation of Indigenous people | Not mentioned | Commissioner meets with Indigenous community members on their country and expects the evaluators to do the same | Commissioner meets with Indigenous community members on their country, on Indigenous terms and in an authentic way |
| Capability building | Commissioner does not expect capacity building to be part of the evaluation. Any appearance of capability building is likely to be tokenistic without any sustainability; black cladding | Varies from tokenistic without any sustainability; black cladding to genuine capability building | Capability is key element of co-design between Commissioner and Indigenous community. Commissioner expects the same from the evaluators | Language encompasses capacity building and capability building as well as empowerment with an equity focus | Language used is capability development and not capacity-building Commissioning of the evaluation prioritises Indigenous capability development (cornerstone) |
| Learning by all stakeholders | Extractive; commissioner does not perceive that learning needs to occur by them or the evaluator | Can occur but often does not depending on level of participation and cultural capability | Key stakeholder learning is key element of co-design | Reflective commissioner; constantly trying to improve the commissioning process | Reflective commissioner; constantly trying to improve the commissioning process for the benefit of the local community through two way learning |
